# Value of inversion imaging to diagnosis in differentiating malignant from benign breast masses

**DOI:** 10.1186/s12880-023-01164-6

**Published:** 2023-12-08

**Authors:** Na Li, Zhongguang Hou, Jiajia Wang, Yu Bi, Xiabi Wu, Yunyun Zhan, Mei Peng

**Affiliations:** Department of Ultrasound, The Second Affiliated Hospital of Anhui Medical University, Economic and Technological Development Zone, No.678, Furong Road, Hefei, Anhui China

**Keywords:** Breast masses, Inversion imaging, Diagnosis, Ultrasound, Breast imaging reporting and Data System

## Abstract

**Background:**

We aimed to evaluate the added value of inversion imaging in differentiating between benign and malignant breast masses when combined with the Breast Imaging Reporting and Data System (BI-RADS).

**Methods:**

A total of 364 patients with 367 breast masses (151 benign and 216 malignant) who underwent conventional ultrasound and inversion imaging prior to breast surgery were included. A 5-point inversion score (IS) scale was proposed based on the masses’ internal echogenicity and distribution characteristics in the inversion images. The combination of IS and BI-RADS was compared with BI-RADS alone to evaluate the value of inversion imaging for breast mass diagnosis. The diagnostic performance of the BI-RADS and its combination with IS for breast masses were analyzed using area under the receiver operating characteristic curve (AUC), accuracy, sensitivity, specificity, positive predictive value (PPV), and negative predictive value (NPV).

**Results:**

The IS for malignant breast masses (3.96 ± 0.77) was significantly higher than benign masses (2.58 ± 0.98) (*P* < 0.001). The sensitivity, specificity, accuracy, PPV, and NPV of BI-RADS were 86.1%, 81.5%, 84.2%, 86.9%, and 80.4%, respectively, and an AUC was 0.909. By compared with BI-RADS, 72 breast masses were downgraded from suspected malignancy to benign, and 6 masses were upgraded from benign to suspected malignancy. Thus, the specificity was increased from 81.5 to 84.8%, it allows 72 benign masses avoid biopsy.

**Conclusion:**

The combination of inversion imaging with BI-RADS can effectively improve the diagnostic efficacy of breast masses, and inversion imaging could help benign masses avoid biopsy.

## Background

 According to the International Agency for Research on Cancer, breast cancer is the most prevalent cancer and the leading cause of death among women worldwide [1–3]. Breast cancer is a growing concern, and the age of its onset is decreasing. Five-year survival rates differ significantly between carcinoma in situ and invasive breast cancer [4, 5]. Therefore, early diagnosis and treatment are key to improving the survival and quality of life of patients with breast cancer [6, 7].

Ultrasonography (US) is an important tool for the diagnosis of breast cancer. Currently worldwide, the US evaluation criteria for breast masses are based on the fifth edition of the Breast Imaging Reporting and Data System (BI-RADS) proposed by the American College of Radiology in 2013 [8]. A BI-RADS category 4 breast mass has wide malignant risk range (2–95%), and images of benign and malignant masses rated as BI-RADS 4 are challenging to distinguish [9–11]. The BI-RADS with US points standardized terminology to describe breast mass US features, and the criteria have been given that emphasize mass shape, margin, orientation, and internal characteristics, posterior features, and the associated features. However, just 2 -10% of the masses in BI-RADS category 4 A are malignant, which leads to a large number of benign patients were received biopsy [8]. Thus, improving the diagnosis of breast masses by US remains a hot issue.

Inversion imaging is a newly developed three-dimensional (3D) US post-processing technique that shows anechoic cystic structures in the region as visible, while the solid gray-scale parts become anechoic [12–15]. The principle of inversion imaging is converting images with hypoechoic as the main background to images with white region as the main background, thereby solving the limitation of low resolution of image details in hypoechoic backgrounds and the inability to recognize the grayscale of cells and tissues with small acoustic impedance differences. Previously, 3D inversion imaging technology was used in the obstetric field, which can significantly improve the diagnostic rate of fetal hydrocephalus, foramina malformation and urinary tract abnormalities [12–15]. Currently, the high frequency linear 3D probes were applied in clinical practice, it is possible to perform inversion imaging of breast masses [16–18]. So far, to the best of our knowledge, there was no study applying inversion imaging to solid masses, especially breast masses.

We proposed the hypothesis that inversion imaging could improve the diagnostic efficiency of breast masses by providing internal structural information according to inversion imaging as the adding value of BI-RADS. In this study, we aimed to analyze inversion imaging of breast masses to investigate its diagnostic effect and evaluate the added value of inversion imaging combined with the BI-RADS of breast masses.

## Materials and methods

The ethics committee of the Second Affiliated Hospital of Anhui Medical University approved this prospective study (SL-YX2022-015), and informed consents were obtained from all patients.

### Study population

**Fig. 1:**
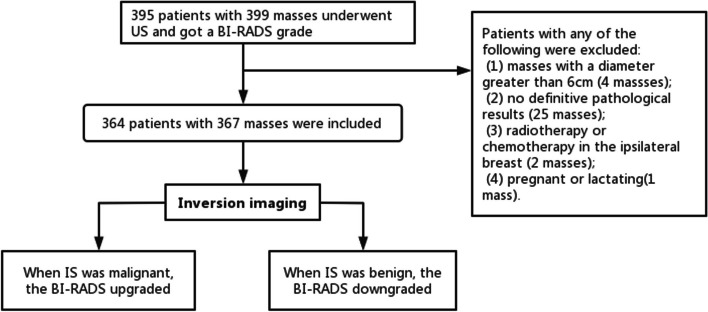
Flow chart of the study

This study was conducted between July 2021 and September 2022. The inclusion criteria were as follows: (a) patients who had breast mass detected by US and (b) patients who was over 18 years old. Exclusion criteria were as follows: (a) a mass with a diameter greater than 6 cm; (b) no definite pathological results; (c) underwent radiotherapy or chemotherapy in the ipsilateral breast before this study; and (d) patients who were pregnant or lactating. The study flow chart was shown in Fig. 1.

### Image acquisition and analysis: US and IS

All examinations were performed using a Samsung RS85A US system (Samsung Madison Ltd., Seoul, South Korea). A 3-12 MHz linear probe was used for US, and a linear 3D volume probe was used for inversion imaging. Images were obtained by two radiologists with over 10 years of experience in US breast imaging. The patients were maintained in a supine position, with full exposure of the breast.

At the first, a US examination was performed using the BI-RADS classification system to evaluate the US features of the breast mass, including shape, margin, orientation, and internal characteristics, posterior features, and the associated features.

The inversion imaging was performed using the linear 3D volume probe. The probe was kept perpendicular to the target lesion, the coronal image of the lesion was reconstructed, the inversion imaging mode was selected based on the coronal plane, the IS images of the maximum coronal, central coronal, and marginal sections of the target lesion were obtained, and the designated range for reverse imaging was selected. The optimal section was observed and selected to avoid calcification and necrosis areas as much as possible. Based on the initial imaging clarity, we determined that the sampling width was 0.5 mm, the threshold was set to 0, the total gain default was 50, and the imaging angle was 0. All the images were obtained under these conditions.

**Fig. 2:**
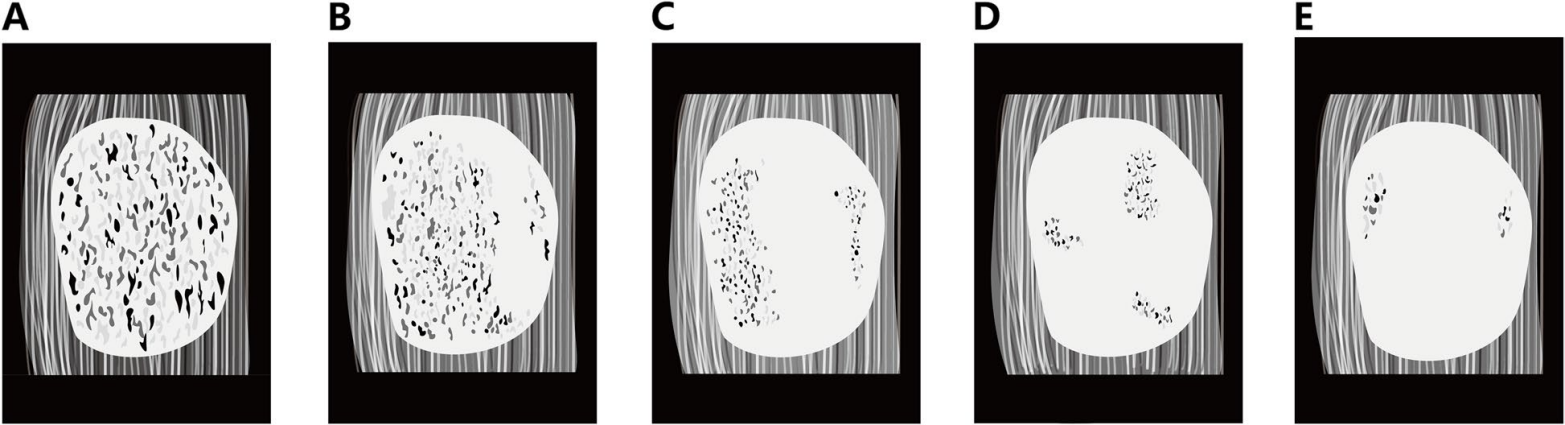
Representative images of the masses’ general appearance by inversion score.  Inversion imaging of **A**: 1 point, **B**: 2 points, **C**: 3 points, **D**: 4 points, and **E**: 5 points. The white region(defined as “Dense Threshold Region”) indicates the breast mass region on inversion images, and the black-white-gray interlaced region (defined as “Non-dense Threshold Region” ) represents the internal acoustic impedance difference

By analyzing the acoustic image characteristics of breast masses in inversion mode, we proposed a five-point scale of IS criteria, which was based on the extent of the distribution of black-white-gray interlaced region (defined as “Non-dense Threshold Region (NTR)”) within the images (Fig. 2). Score 1: a mass diffuse distribution of NTR within the mass. Score 2: a mass where more than two-thirds of the area was NTR. Score 3: a mass where between one-third and two-thirds of the area was NTR. Score 4: a mass where less than one-third of the area was NTR. Score 5: a mass with less than three NTRs. When the score was above the cutoff value (described below), the breast mass was categorized as malignant, and if equal to or below the cutoff point, as benign.

### Combined diagnosis

We divided the data into two groups for analysis and comparison: BI-RADS and BI-RADS + IS. In the BI-RADS + IS group, the original BI-RADS was obtained based on US, and when the breast mass was determined as malignant by IS, the original BI-RADS was upgraded by one; otherwise, it was downgraded by one. However, the masses primarily determined as BI-RADS category 3 were not downgraded, and the masses primarily determined as BI-RADS category 5 were not upgraded.

#### Statistical analysis

Quantitative data are expressed as means and standard deviations, and non-normal quantitative data are expressed as medians. The best cutoff value for the IS was determined using the receiver operating characteristic (ROC) curve and Youden index analysis. To explore whether the combination of US and IS facilitates the diagnosis of the breast mass, AUC, sensitivity, specificity, accuracy, PPV, and NPV represented the diagnostic value of the three methods, and a McNemar’s test was used to compare the differences in diagnostic efficacy between the groups. Statistical analysis was performed using IBM SPSS Statistics for Windows, Version 26.0 (IBM Corp., Armonk, NY, USA). Differences with *P* < 0.05 were considered statistically significant.

## Results

### Pathology results

Based on the inclusion and exclusion criteria, this study included 364 patients (mean age, 47.0 ± 13.7 years) with 367 breast masses, including 216 malignant (58.9%) and 151 benign (41.1%) masses. The patients with malignant masses were older than the patients with benign masses (53.6 ± 11.6 vs. 37.6 ± 11.5 years; *P* < 0.001) and had larger lesions (2.8 ± 1.3 vs. 1.9 ± 1.0 cm; *P* < 0.001). The final pathological diagnoses of these masses were presented in Table 1. The main one of malignant mass was invasive ductal carcinoma (152 masses, 70.4%). Fibroadenomas were the most prevalent benign lesion (77 masses, 60.0%).

**Table 1:** Histological diagnosis of masses confirmed by pathology and the inversion score of each classification

Malignant			Benign		
Histologic features	N (%)	IS (mean ± SD)	Histologic features	N (%)	IS (mean ± SD)
Invasive ductal carcinoma	152 (70.4)	4.0 ± 0.8	Fibroadenoma	77 (60.0)	2.5 ± 0.9
Ductal carcinoma in situ	47 (21.8)	3.9 ± 0.7	Mammary adenosis	55 (36.4)	2.5 ± 1.0
Mucous carcinoma	5 (2.3)	4.0 ± 0.7	Intraductal papilloma	6 (4.0)	2.5 ± 1.1
Well-differentiated neuroendocrine carcinoma	4 (1.9)	3.5 ± 0.6	Usual ductal hyperplasia	5 (3.3)	2.6 ± 1.1
Lobular carcinoma	3 (1.4)	4.3 ± 0.6	Benign phyllodes tumor	4 (2.6)	3.5 ± 0.6
Basaloid carcinoma	3 (1.4)	3.7 ± 0.6	Mastitis	4 (2.6)	3.3 ± 1.0
Solid papillary carcinoma	1 (0.5)	4.0			
Diffuse large-cell B lymphoma	1 (0.5)	3.0			

*N *Number, *SD *Standard deviation

#### Diagnostic efficacy of BI-RADS and IS alone

**Fig. 3:**
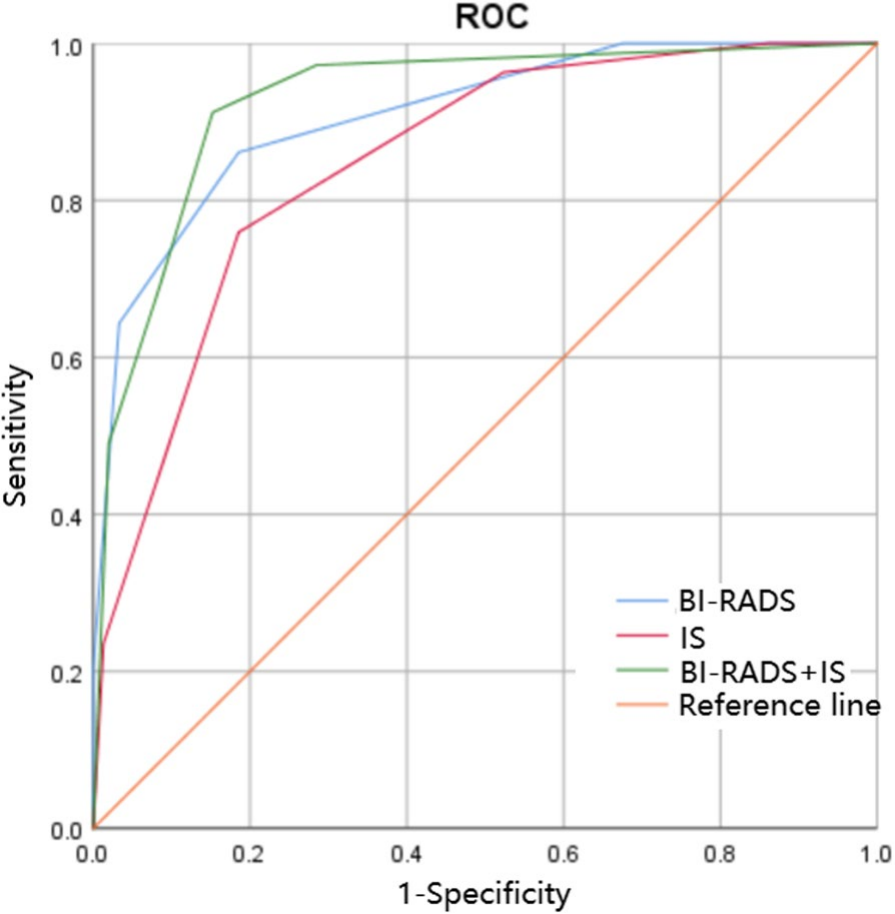
Receiver operating characteristic curve. ROC of BI-RADS, IS and BI-RADS + IS to differentiate malignant from benign breast masses

**Fig. 4:**
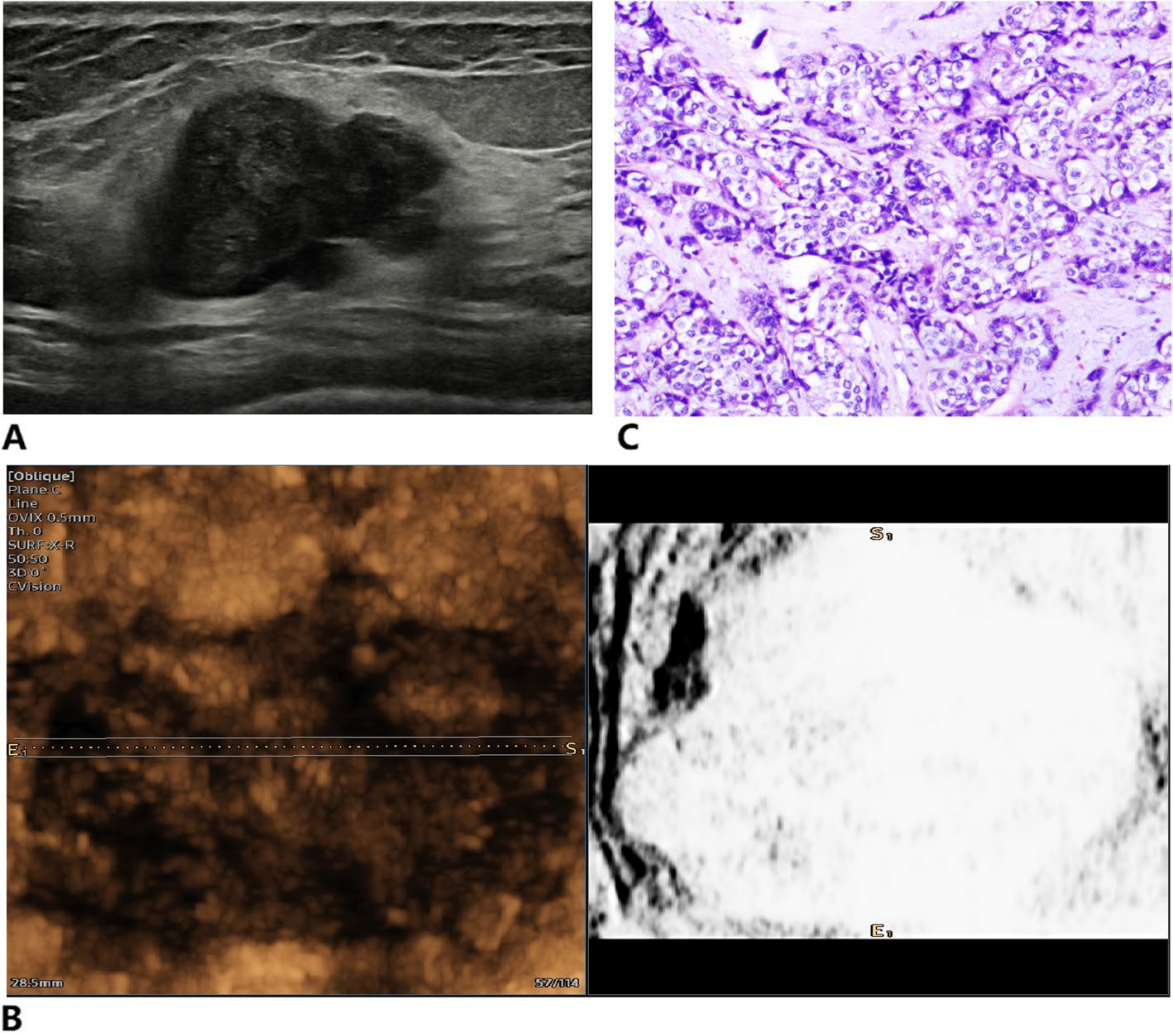
Infiltrating ductal carcinoma of the right breast. **A** Two-dimensional ultrasound image was classified the breast mass as BI-RADS 4A. **B** Inversion imaging image showing a white mass with less than three NTRs, and the point of inversion was 5. **C** Pathological section showing that the mass was an invasive ductal carcinoma

**Fig. 5:**
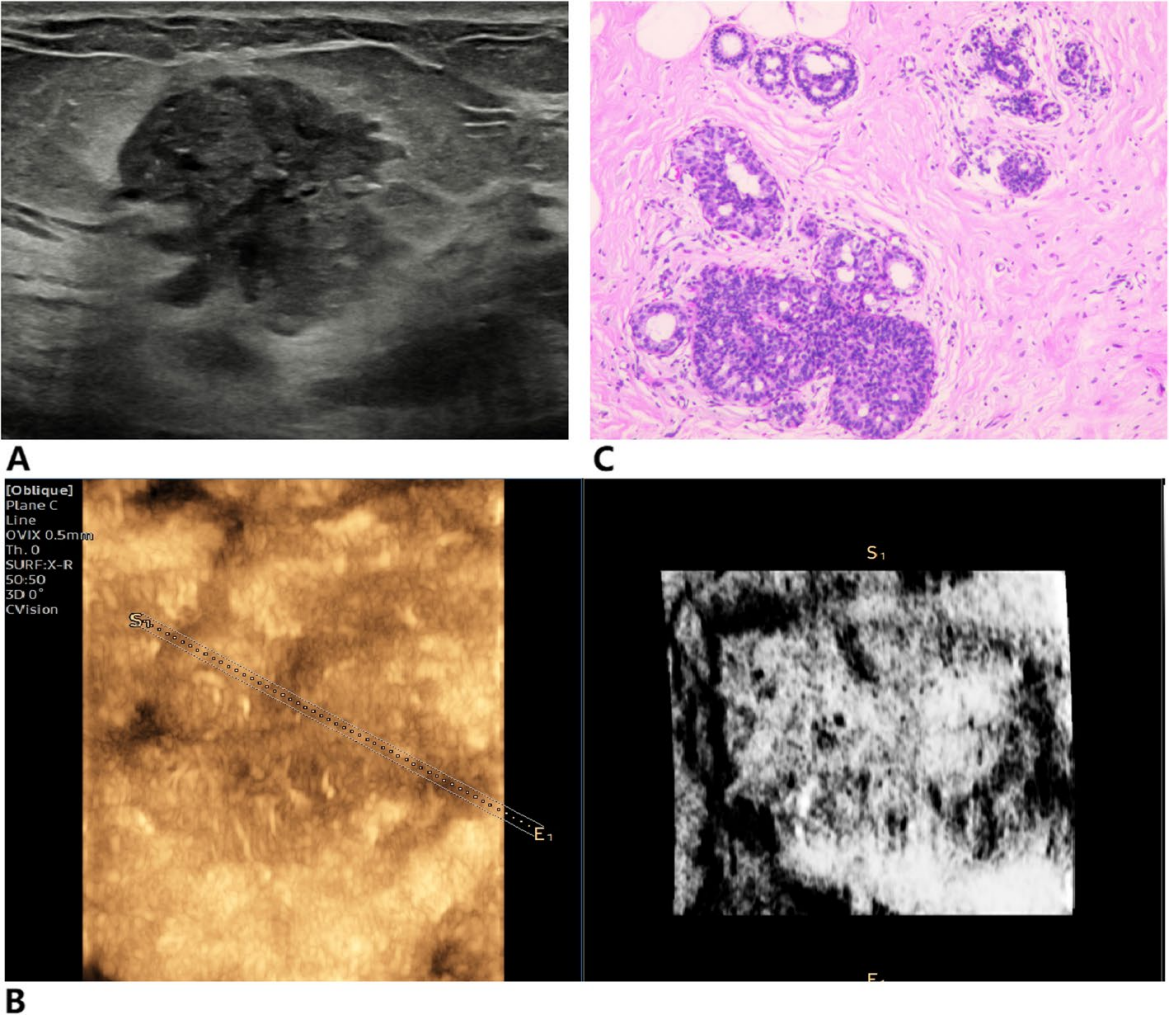
Mammary adenosis of the left breast. **A** BI-RADS 4B breast mass in ultrasound image. **B** Inversion imaging showing diffuse distribution of NTR within the mass, the inversion score was 1. **C** Pathological section showing that the mass was a mammary adenosis with a small amount of surrounding duct dilation

**Fig. 6:**
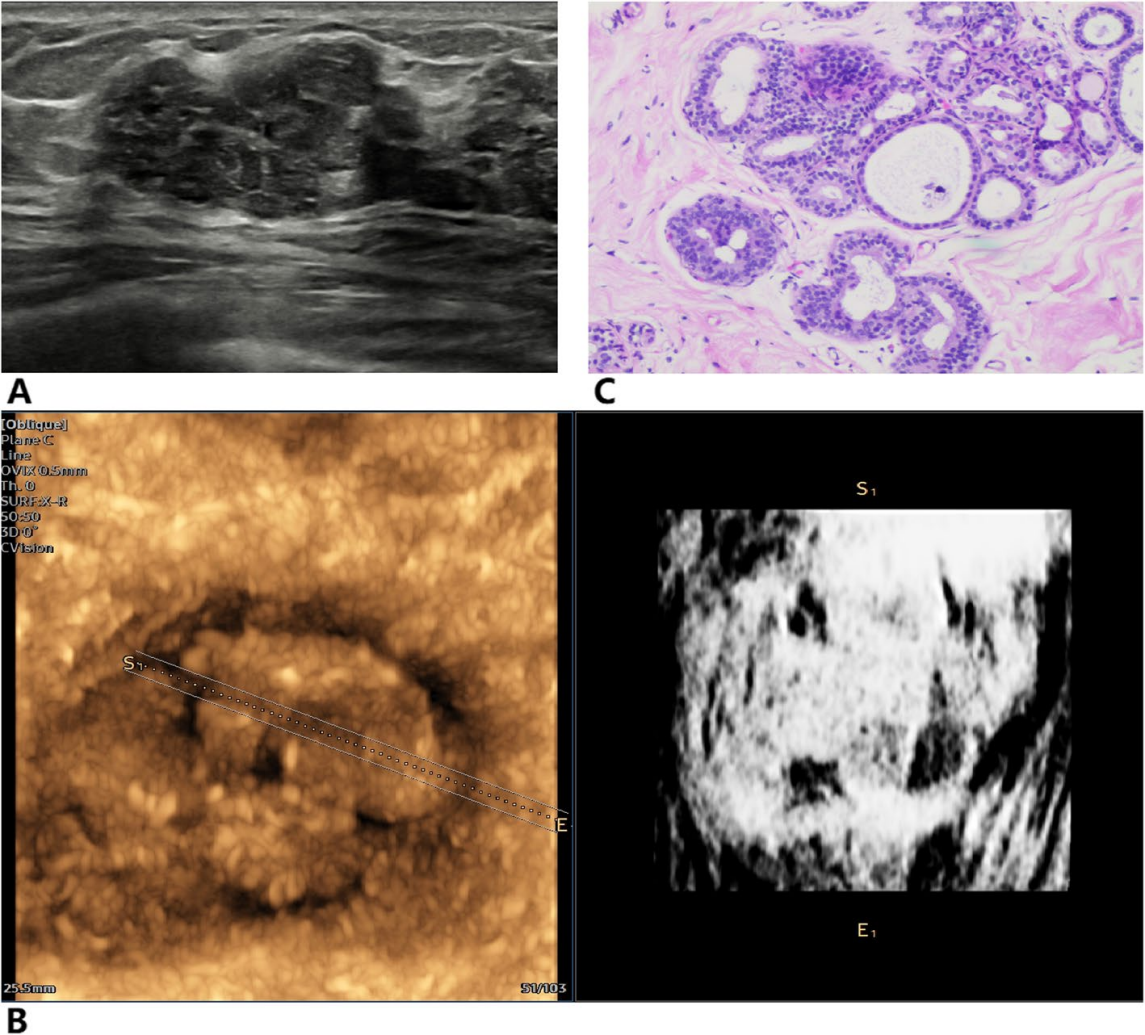
Fibroadenoma of the right breast. **A** Two-dimensional ultrasound image indicating that the mass was classified as BI-RADS 4A. **B** Inversion imaging image showing that more than two-thirds of the area within the mass was NTR, the inversion score was 2. **C** Pathological section showing that the mass was a fibroadenoma

**Fig. 7:**
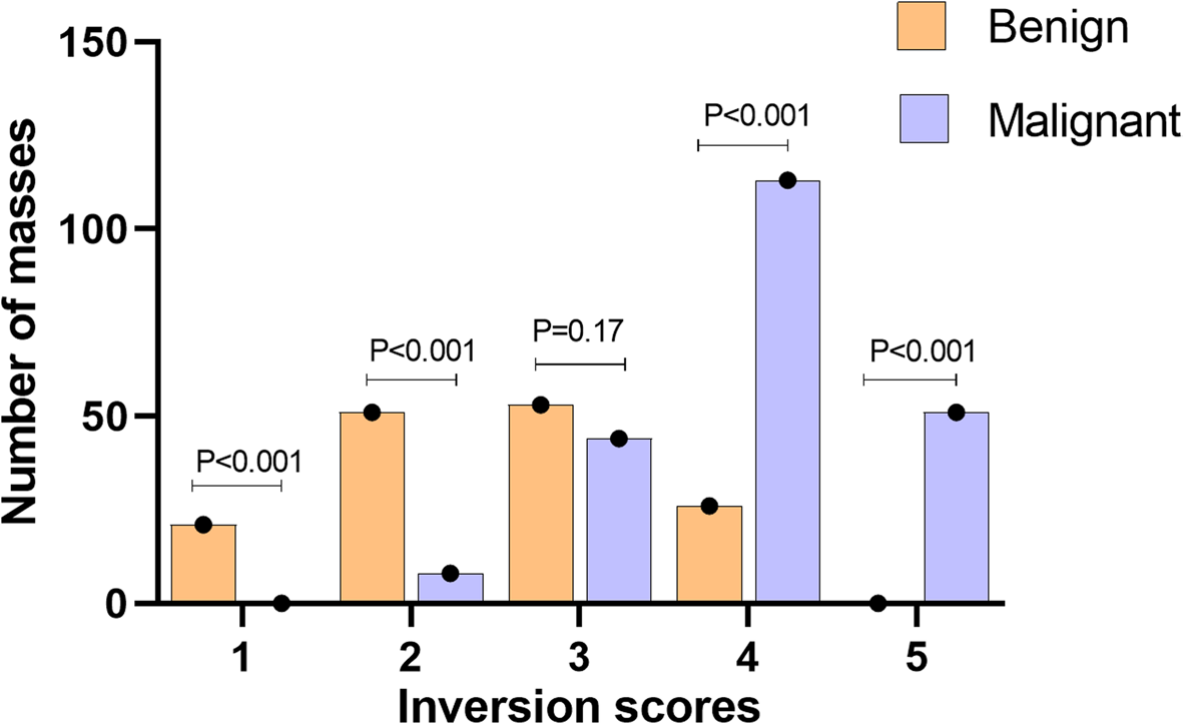
Bar graph demonstrating the comparison of the number of masses with different inversion scores in the benign and malignant group

The cutoff value for the differentiation of malignant masses from benign masses in IS was 3.50, with an AUC of 0.849 (Fig. 3). Representative IS and BI-RADS images were shown in Figs. 4, 5 and 6. The diagnostic efficacy of BI-RADS and IS were shown in Table 2. The IS of the malignant masses was significantly higher than that of the benign masses (3.96 ± 0.77 vs. 2.58 ± 0.98, respectively, *P* < 0.001). The comparisons of the number of benign and malignant masses in each IS were shown in Fig. 7. The IS values increased with the lesion’s BI-RADS. The mean IS values of benign/malignant masses for each category were shown in Table 3. Additionally, there were significant statistical differences in the distribution of benign and malignant breast masses in the IS and BI-RADS classification (all *P* < 0.001).

**Table 2:** Comparison of diagnostic method efficacy by mass-size group and among the entire cohort

Diagnostic Method	AUC	Accuracy, %	Sensitivity, %	Specificity, %	PPV, %	NPV, %
BI-RADS	0.909 (0.880–0.938)	84.2 (80.5–87.9)	86.1 (82.6–89.6)	81.5 (77.6–85.4)	86.9 (83.5–90.4)	80.4 (76.3–84.5)
IS P^*^	0.849 (0.809–0.889) < 0.001	78.2 (74.0–82.4) < 0.001	75.9 (71.5–80.3) < 0.001	81.5 (77.6–85.4) > 0.05	85.4 (81.8–89.0) < 0.001	70.3 (65.6–75.0) < 0.001
BI-RADS + IS P^*^	0.927 (0.899–0.956) < 0.001	88.6 (85.4–91.8) < 0.001	91.2 (88.3–94.1) < 0.001	84.8 (81.1–88.5) < 0.001	89.5 (86.3–92.7) < 0.001	87.1 (83.6–90.6) < 0.001

^*^Comparison with BI-RADS

**Table 3:** Distribution of breast masses and comparison of inversion score

Diagnostic method	All masses(N)	Malignant(N)	Benign(N)	IS (mean ± SD)	P^*^
BI-RADS	367	216	151	3.39 ± 1.09	< 0.001
3	49	0	49	2.67 ± 0.85	< 0.001
4 A	104	30	74	2.78 ± 1.17	< 0.001
4B	70	47	23	3.67 ± 0.91	< 0.001
4 C	100	95	5	3.95 ± 0.86	< 0.001
5	44	44	0	3.93 ± 0.63	< 0.001
IS	367	190	177		< 0.001
BI-RADS + IS	367	220	147		< 0.001
IS (mean ± SD)	3.39 ± 1.09	3.96 ± 0.77	2.58 ± 0.98		< 0.001

^*^Comparison between benign and malignant

#### Diagnostic efficacy of BI-RADS combined with IS

**Fig. 8:**
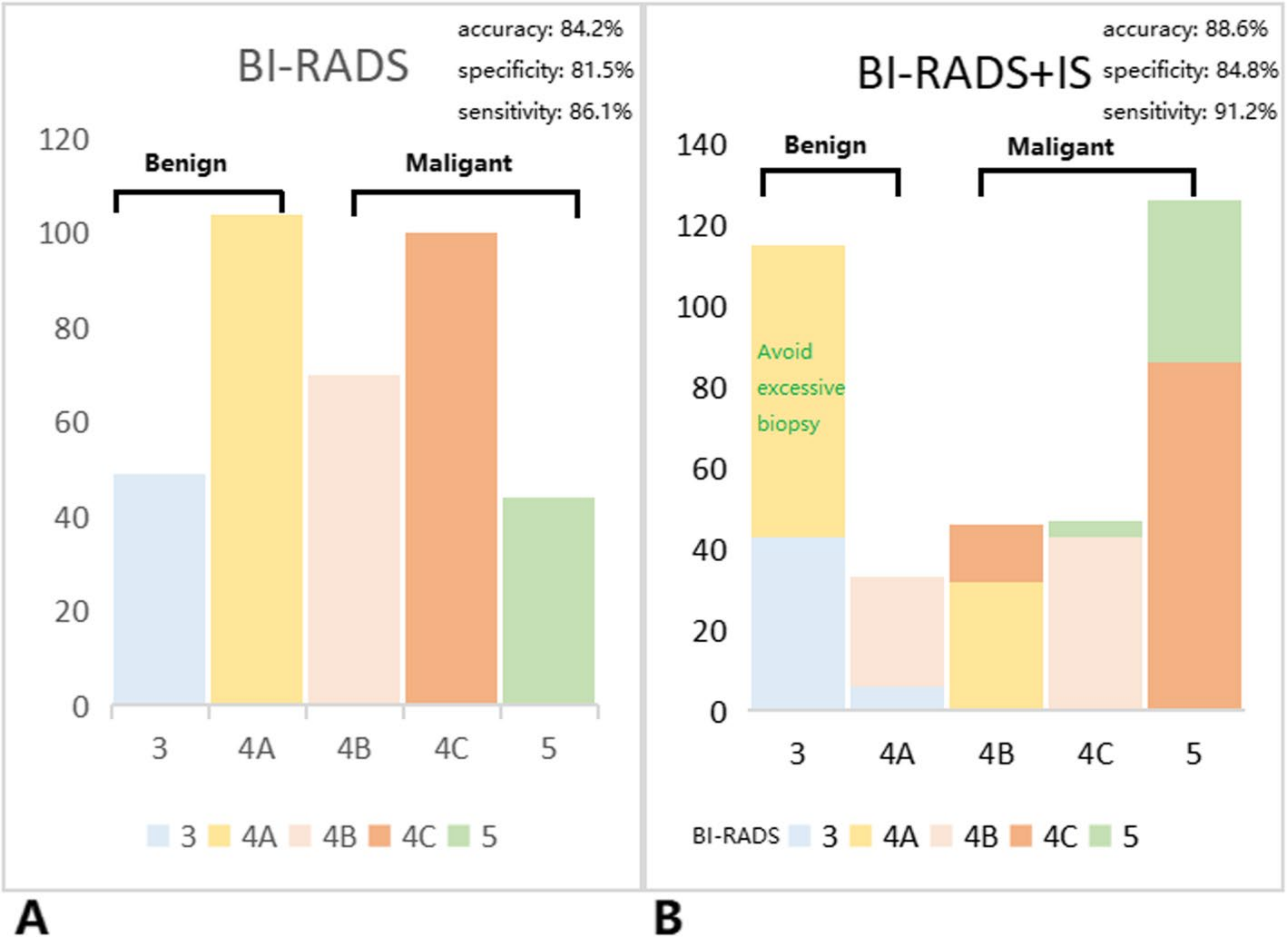
Bar graph demonstrating the distribution of breast masses of BI-RADS (**A**) and BI-RADS+IS (**B**)

119 benign masses were correctly downgraded, including 72 masses avoided biopsy by downgrading from BI-RADS category 4 A to BI-RADS category 3. Additionally, 136 malignant masses were successfully upgraded. The specific upgraded and downgraded situation of combined diagnosis was shown in Fig. 8.

The sensitivity, specificity, accuracy, PPV, and NPV of the combined diagnostic approach (91.2%, 84.8%, 88.6%, 89.5, and 87.1%, respectively) were significantly higher and the AUC was significantly larger (0.927 vs. 0.909; *P* < 0.001) than the BI-RADS group (Table 2; Fig. 3).

## Discussion

To the best of our knowledge, this study is the first to apply inversion imaging to masses in the breast and differentiate masses according to their internal acoustic impedance differences. We proposed a 5-point scale of IS criteria to semi-quantitatively reflect the acoustic impedance difference between masses based on their internal echo and distribution characteristics by inversion imaging. In addition, after adding inversion imaging based on BI-RADS, our combined BI-RADS + IS has higher diagnostic efficiency compared with BI-RADS alone. Our results show that inversion imaging has value in evaluating benign and malignant breast masses.

Both malignant and benign solid breast masses are hypoechoic in US images, and it is difficult to identify the difference between masses by their internal gray level difference when the gray level difference is beyond the range that human vision can recognized [8, 19]. Inversion imaging is a post-processing technique, can invert the grayscale of a two-dimensional ultrasound image, which can better reflect this gray scale difference and show the acoustic impedance difference within the mass more clearly [20, 21]. In inversion imaging, the more white-regions of inversion imaging of the mass, it be supposed to more likewise malignancy, since such image present low different in acoustic impedance. Pathology suggests that malignant tissues with predominantly solid or syncytial cell-like infiltrative growth and a lack of interstitial space have a more homogeneous structure, resulting in a more uniform and smaller acoustic impedance difference [22, 23]. Therefore, the benign masses overall image is blacker and less homogeneous than malignant masses during inversion imaging.

Our study showed that the IS of malignant masses was significantly higher than that of benign masses, indicating that inversion imaging is beneficial for the differential diagnosis of benign and malignant breast masses. The AUC of this diagnosis was 0.849, which supports the hypothesis that inversion imaging has a better diagnostic value for benign and malignant breast masses and can be applied in clinical examinations. However, the cyst shows anechoic in conventional ultrasound, but because of its small internal acoustic impedance difference, it shows white and overall uniform distribution in inversion imaging, which is easily misdiagnosed as malignant [24]. Therefore, in the clinical application of inversion imaging, it must be performed on the basis of conventional US to recognize the solid mass and reduce the false positive rate.

The US BI-RADS is a standardized method for assessing the degree of benignity and risk of breast masses [8]. The BI-RADS also has a certain rate of misdiagnosis because of the overlap of US imaging signs of some benign and malignant breast masses [25, 26]. Inversion imaging combined with the BI-RADS is based on the BI-RADS and highlights the information of the acoustic impedance difference within the solid masses simultaneously in a semi-quantitative manner. The combination of the two methods resulted in an improvement in AUC, sensitivity, specificity, accuracy, and PPV compared to the application of BI-RADS alone. The combination of these two methods has a synergistic effect and can improve the efficacy of the diagnosis of benign and malignant solid breast masses.

After using the combined diagnostic method, among the 138 breast masses that were downgraded, 72 of them were downgraded from BI-RADS category 4 A to BI-RADS category 3, thus effectively avoiding unnecessary biopsies of benign masses and reducing patients’ pain and uneasiness; 19 of these were incorrectly downgraded malignant lesions, most of which were invasive ductal carcinomas, non-specific type, probably with tissue necrosis inside the masses resulting in internal echogenicity that were not uniform, and the acoustic impedance difference was large, leading to misclassification. However, most of these masses were diagnosed as BI-RADS category 4 C or BI-RADS category 5 by US, and after being downgraded by one grade of combined diagnosis, they were mostly BI-RADS category 4 C or BI-RADS category 4B, the patients still met the biopsy criteria, which prevented the delay of the diagnosis. Among the 153 breast masses that were upgraded, there were 10 cases of incorrectly upgraded benign masses. There were four cases of mammary adenosis, three cases of ductal hyperplasia, two cases of benign phyllodes tumor, and one case of intraductal papilloma, probably due to the exuberant growth of internal tissues and rapid short-term growth of the same type of cells, resulting in a small acoustic impedance difference in most of its internal regions that caused an increased IS and misclassification.

This study has some limitations. Although we used the semi-quantitative 5-point evaluated the internal structure of breast masses and assist in diagnosing the benign and malignant of breast masses, but it still has an extent subjectivity when we read the inversion imaging, which only improved by continuously training diagnostic operators to read the 5-point method in the later stage. Meanwhile, this was a single-center study, we did not perform a subgroup analysis of the relationship between various pathological types and IS. A larger sample size would be needed for such an analysis because of the relatively small number of cases available in each subgroup. As all US images were obtained using the same instrument, the applicability of our findings to other devices could not be confirmed.

## Conclusion

In summary, the addition of inversion imaging to BI-RADS can improve diagnostic efficacy of breast masses, and inversion imaging was expected to be used as a new useful examination in the future to determine the benign and malignancy of masses.

## Data Availability

The datasets used during the current study are available from the corresponding author on reasonable request.

## Abbreviations

BI-RADS: Breast Imaging Reporting and Data System
IS: Inversion score
AUC: Area under the receiver operating characteristic curve
PPV: Positive predictive value
NPV: Negative predictive value
US: Ultrasonography
3D: Three-dimensional
NTR: Non-dense Threshold Region
ROC: Receiver operating characteristic
N: Number
SD: Standard deviation
